# Miscellany

**Published:** 1893-09

**Authors:** 


### Miscellany.

College of Physicians of Philadelphia.—The William F. Jenks memorial prize. The third triennial prize of $500, under the deed of trust of Mrs. William F. Jenks, will be awarded to the author of the best essay on Infant Mortality During Labor, and Its Prevention.

The conditions annexed by the founder of this prize are: That the "prize or award must always be for some subject connected with obstetrics, or the diseases of women, or the diseases of children;" and that "the trustees, under this deed for the time being, can, in their discretion, publish the successful essay, or any paper written upon any subject for which they may offer a reward, provided the income in their hands may, in their judgment, be sufficient for that purpose, and the essay or paper be considered by them worthy of publication. If published, the distribution of said essay shall be entirely under the control of said trustees. In case they do not publish the said essay or paper, it shall be the property of the College of Physicians of Philadelphia.

The prize is open for competition to the whole world, but the essay must be the production of a single person.

The essay, which must be written in the English language, or, if in a foreign language, accompanied by an English translation, should be sent to the College of Physicians of Philadelphia, Pa., U. S. A., before January 1, 1895, addressed to Horace Y. Evans, M. D., Chairman of the William F. Jenks Prize Committee.

Each essay must be typewritten, distinguished by a motto, and accompanied by a sealed envelope bearing the same motto and containing the name and address of the writer. No envelope will be opened except that which accompanies the successful essay.

The committee will return the unsuccessful essays if reclaimed by their respective writers, or their agents, within one year.

The committee reserves the right not to make an award if no essay submitted is considered worthy of the prize.

JAMES V. INGHAM,
*Secretary of the Trustees.*

---

THE PAN-AMERICAN MEDICAL CONGRESS—SECTION ON DISEASES OF CHILDREN.—The organization of this section is complete, and the work of arranging a program is well advanced. Numerous valuable papers have been promised, and the success of the meetings is assured. Physicians interested in diseases of children are cordially invited to attend these meetings, which give promise of great interest both to the specialist and general practitioner. Any American physician desiring to read a paper will please communicate at once with the Secretary, who will be pleased to furnish all needed information.

*Executive President*—Dr. John M. Keating, Colorado Springs, Colorado.

*Secretaries*—Dr. F. M. Crandall (English-speaking), No. 113 W. Ninety-fifth street, New York, N. Y.; Dr. Damaso Lainé (Spanish-speaking), Media, Pa.

*Honorary Presidents*—Dr. S. S. Adams, Washington ; Dr. A. D. Blackader, Montreal, Canada ; Dr. Samuel C. Busey, Washington ; Dr. Charles Warrington Earle, Chicago ; Dr. F. Forchheimer, Cincinnati ; Dr. L. Emmet Holt, New York ; Dr. A. V. Meigs, Philadelphia ; Dr. W. P. Northrup, New York ; Dr. J. O'Dwyer, New York ; Dr. C. I. Putnam, Boston ; Dr. T. M. Rotch, Boston ; Dr. J. Lewis Smith, New York ; Dr. Louis Starr, Philadelphia ; Dr. J. E. Winters, New York ; Dr. Jesus Valenzuela, City of Mexico, Mexico ; Dr. I. N. Love, St. Louis, Mo.

*Advisory Council*—Dr. William D. Booker, Baltimore ; Dr.

Augustus Caillé, New York; Dr. Henry D. Chapin, New York; Dr. J. P. Crozer Griffith, Philadelphia; Dr. M. P. Hatfield, Chicago; Dr. Thomas S. Latimer, Baltimore; Dr. J. H. Ripley, New York; Dr. August Seibert, New York; Dr. Charles W. Townsend, Boston; Dr. Jerome Walker, Brooklyn; Dr. William Perry Watson, Jersey City.

---

### PAN-AMERICAN MEDICAL CONGRESS.

*Committee of Arrangements.*—Samuel S. Adams, M. D., Chairman; J. R. Wellington, M. D., Secretary; G. L. Magruder, M. D., Treasurer.

*Executive Committee.*—Dr. Samuel S. Adams, Chairman; Surgeon-Generals Geo. M. Sternberg, U. S. A.; J. Rufus Tyron, U. S. N.; Walter Wyman, U. S. M. H. S.; Drs. S. C. Busey, G. Wythe Cook, Carl H. A. Kleinschmidt, H. L. E. Johnson, Llewellyn Eliot, H. H. Barker, C. W. Richardson, W. Sinclair Bowen, Geo. S. Ober, G. L. Magruder, J. R. Wellington, and John R. Walton, D. D. S.

### SUB-COMMITTEES.

*Reception.*—Dr. S. C. Busey, Chairman; Surgeon-Generals Geo. M. Sternberg, U. S. A.; J. Rufus Tyron, U. S. N.; Walter Wyman, U. S. M. H. S.; Drs. J. Ford Thompson, Charles Hagner, Louis Mackall, J. Taber Johnson, T. Morris Murray, G. Byrd Harrison, and Jos. H. Byran.

*Entertainments.*—Dr. G. Wythe Cook, Chairman; Drs. G. N. Acker and Thos. E. McArdle.

*Registration.*—Dr. Carl H. A. Kleinschmidt, Chairman; Drs. John S. McLain and Johnson Eliot.

*Railroads.*—Dr. H. L. E. Johnson, Chairman; Drs. E. L. Tompkins and J. Foster Scott.

*Printing.*—Dr. Llewellyn Eliot, Chairman; Drs. Thomas N. Vincent and F. B. Bishop.

*Halls and Exhibits.*—Dr. H. H. Barker, Chairman; Drs. J. T. Winter and C. M. Buchanan.

*Ways and Means.*—Dr. C. W. Richardson, Chairman; Drs. John Van Rensselaer, Wm. Dillenback, Henry B. Deale and Wm. Compton.

*Information.*—Dr. W. Sinclair Bowen, Chairman; Drs. E. Oliver Belt and F. S. Nash.

*Hotels.*—Dr. Geo. S. Ober, Chairman; Drs. Wm. E. Handy and D. O. Leech.

